# Application of Non-Destructive Rapid Determination of Piperine in *Piper nigrum* L. (Black Pepper) Using NIR and Multivariate Statistical Analysis: A Promising Quality Control Tool

**DOI:** 10.3390/foods9101437

**Published:** 2020-10-11

**Authors:** Jong-Rak Park, Hyun-Hee Kang, Jong-Ku Cho, Kwang-Deog Moon, Young-Jun Kim

**Affiliations:** 1 School of Food Science and Biotechnology, Kyungpook National University, Daegu 41566, Korea; jongraki@knu.ac.kr (J.-R.P.); kdmoon@knu.ac.kr (K.-D.M.); 2 Department of Food Science and Technology, Seoul National University of Science and Technology, Seoul 01811, Korea; khh266900@seoultech.ac.kr; 3 Nanomarkers Co. Ltd., Seongnam 13595, Korea; mkt@pointer.kr

**Keywords:** near infrared spectroscopy, piperine, *Piper nigrum* L., eco-friendly analysis, chemometric modeling

## Abstract

Piperine is a bioactive alkaloid compound which provides a unique spicy flavor derived from plants of the *Piper nigrum* L. Black pepper (*n* = 160) collected from Vietnam was studied using non-destructive near infrared spectroscopy (NIRS). The spectral acquisition ranged from 1100 to 2500 nm, and a chemometrics analysis program was performed to quantify the piperine contents. High performance liquid chromatography (HPLC) analysis was carried out to develop a chemometric model based on reference values. The black pepper samples were divided into two groups used for calibration (*n* = 120) and prediction (*n* = 40) sets. The optimum calibration model was developed by pretreatment of the spectra. The analyses results based on the prediction samples included a coefficient of determination (*R*^2^) of 0.914, a root mean square error of prediction (RMSEP) and a standard error of prediction (SEP) of about 0.220 g/100 g, and a ratio performance to deviation (RPD) value of 3.378 regarding the partial least square (PLS) regression model, and an *R*^2^ of 0.921, an RMSEP and SEP of 0.210 g/100 g, and an RPD of 3.571, with respect to the principal components (PC) regression model. These results indicate that NIRS can be applicable as a control, or as an alternative rapid and effective method to quantify piperine in *P. nigrum* L.

## 1. Introduction

Pepper (*Piper nigrum* L.) is a fruit of the family *Piperaceae* and native to southern India. As of the year 2018, the production of pepper (*Piper* spp.) was 690,698 tons all over the world. Production increased by 6.2% annually from 2012 to 2018. Vietnam is the largest producer, accounting for 34% of the global production, followed by Brazil and Indonesia, accounting for 13.1% and 11.5%, respectively [1]. Pepper is a typical spice that has been used for cooking since ancient times, and has a unique spicy taste that adds flavor and removes odor when cooking. Black pepper is the peppercorns dried and crushed with the flesh, and white pepper is the seeds of the ripe peppercorns dried after peeling the skin [2]. Piperine is an important bioactive compound and a major alkaloid component of pepper. The effects of piperine include immunomodulatory, anti-carcinogenic [3], antimicrobial [4], anti-inflammatory [5,6], anti-cancer [7,8] and anti-ulcer activities [9].

Traditionally, methods for analyzing the piperine content of pepper have included UV spectrophotometry [10], TLC-UV densitometry [11], high-performance thin-layer chromatography (HPTLC) [12], HPLC [13,14] and the electrochemical quantification method [15]. When analyzed using HPLC and HPTLC, the piperine content of pepper was reported to be 3% to 6% [16]. Piperine analysis using high performance liquid chromatography (HPLC) requires the pre-treatment of samples, such as extraction, separation and purification. Depending on the proficiency of the tester, it involves processes such as the error calculation of the result value generated in the analysis process, the handling of the solvent used for pretreatment harmful to the human body, the treatment of waste organic solvent and the manipulation of complex instruments. This analysis method is time-consuming, and the results are confirmed through all these processes.

Near infrared spectroscopy (NIRS) is a representative environmentally friendly non-destructive analysis method. When the wavelength of the near infrared region is irradiated to the sample, absorption of the wavelength occurs due to the vibration of molecules in the sample. NIRS measures the physical and chemical properties of a sample by recording changes in the absorbed wavelengths (in nm). The generated spectrum can be correlated with data obtained for the desired component through chemical experiments using chemometric analysis with statistical and mathematical processing. The advantages of NIRS compared to conventional piperine analyses methods include the following: (1) there is no need to extract piperine from the sample (non-destructive analysis), (2) no organic solvent is used for the extraction (eco-friendly), (3) there is a reduced analysis cost due to the omission of the pre-treatment process (cost-effective), (4) it gives highly reliable results due to minimized errors among testers, and (5) it involves a short analysis time compared to HPLC. Due to these advantages, NIR spectroscopy is widely used to analyze foods such as fruits and vegetables [17], cereals [18], bakery [19] and dairy products [20]. In addition, it is applied in the pharmaceutical industry, which requires higher accuracy for the quality control of end products and production lines [21]. Recently, in order to maximize the advantages of NIRS, portable system have also been applied in many fields, such as analyzing the quality of instant green tea [22], the internal quality of citrus fruit [23] and the quality of dairy farm forage [24].

This study aims to verify whether piperine, a representative active ingredient of pepper, can be analyzed using NIRS as a quality control tool combined with chemometric modeling.

## 2. Materials and Methods 

### 2.1. Chemicals and Materials

The black pepper samples (*n* = 160) were imported from Vietnam during 2016–2018 by OTTOGI Sesame Co. (Umseoung, Korea). The samples were placed in a dark space at 22–26 °C prior to analysis, and the collected samples were subjected to quarterly piperine analysis. Samples were powdered with a blender (HR 2860, Philips, Shanghai, China) and transferred with 60 mesh sieves for obtaining a ground powder. An analytical grade reference standard of piperine (purity 98.5%) and citric acid as the mobile phase for HPLC were purchased from Sigma (St. Louis, MO, USA). The methanol and acetonitrile of HPLC grade were supplied by Fisher Scientific (Pittsburgh, PA, USA). Deionized water (18.2 MΩ) was purified using an ultra-pure water system (OmniaTap6, Stakpure, Niederahr, Germany). 

### 2.2. Reference HPLC Method for Analyzing Piperine

The piperine profiling was performed as described below [25,26]. For quantitative analysis, approximately 0.1 g of homogenized sample was placed into a 50 mL conical tube to which 50 mL of methanol was added. The mixture was ultrasonically extracted at 50 °C for 20 min, cooled to 25 °C, and filtered through a 0.45 μm regenerated cellulose membrane syringe filter (Sartorius, Göttingen, Germany). HPLC analyses were carried out using an Agilent 1100 HPLC system (Agilent, Santa Clara, CA, USA) with a diode array detector (DAD, 340 nm). An Eclipse C18 plus column (4.6 × 150 mm, 5 μm, Agilent, Santa Clara, CA, USA) was utilized at 25 °C for piperine quantification. The mobile phase was acetonitrile/1% citric acid (45:55, isocratic). The sample injection volume was 10 μL, and the flow rate was 1 mL per minute with a run time of 20 min. ChemStation software Rev.A.10.02, (Agilent, Santa Clara, CA, USA) was applied for all analytical conditions and the chromatographic data processing. Sample qualification and quantification were conducted using the DAD spectra comparing the peak area of chromatograms analyzed with the external calibration curve from the standard solution (20–500 mg/100 g). The piperine content was represented as g/100 g of ground black pepper sample, and the contents determined by HPLC (as a reference data) were compared to the NIRS measurements (as experimental data). Before performing NIRS analysis, the piperine content in ground black pepper was analyzed using HPLC to obtain the reference data.

### 2.3. NIRS Measurement

The same black pepper sample used for HPLC was used for the NIRS analysis. The NIR equipment was optimized in the reflection mode using a ceramic standard before analysis, and the ground black pepper sample was analyzed in diffuse reflectance mode (model 5000 monochromator, FOSS NIRS Systems Inc., Silver Spring, MD, USA). A small ring cup with a diameter of 50 mm was employed for the sample, and about 3 g of ground black pepper was used for the measurement. The analysis wavelengths ranged between 1100 and 2500 nm, and data were collected from a total of 700 wavelengths at 2 nm intervals. To reduce the noise of each spectrum, 25 scans were averaged with a 2-min measuring time. The laboratory temperature and relative humidity were kept at 22–26 °C and 45–60%, respectively. The NIRS data was collected using WinSIS II software (Foss and Infrasoft International LLC, State College, PA, USA).

#### 2.3.1. Data-Pretreatment

The NIRS spectra data were analyzed by multivariate statistical analysis using the Unscrambler^®^ X, v10.5 (CAMO Software AS, Oslo, Norway). Prior to creating and verifying the chemometrics model, data pretreatment was performed on the obtained NIRS spectrum. Standard normal variate (SNV) is a method that corrects the scattering and dispersion of light in the spectrum and stabilizes the baseline of the spectrum [27]. De-trending (DT) adjusts the change in curvature of the baseline of the SNV that corrects the data by moving the data along the y-axis [28]. Using the Savitzky and Golay smoothing filter, the derivation and smoothing points were adjusted to increase the signal-to-noise ratio, reducing the interference between the medium that disperses light and other materials that absorb light [29].

#### 2.3.2. Chemometrics Development and Evaluating the Prediction Model

The 160 NIRS profiles obtained from the whole samples set were divided into two groups. Group 1 (*n* = 120) was used for calibration and cross-validation, while group 2 (*n* = 40) was used for the prediction to be applied to an optimized chemometrics model. To construct the NIRS prediction calibration model, partial least squares (PLS) and principal component (PC) were applied as regression methods. Cross validation to verify the calibration curves was performed on 20 segments (6 samples per each segment) randomly extracted from the calibration set. The numbers of factors were adapted in an optimization process between over-fitting and under-fitting. The performance of the models was evaluated using the coefficient of determination (*R*^2^), the root mean square error of performance (RMSE), the standard error of calibration (SEC), the standard error of cross-validation (SECV) and the standard error of prediction (SEP), as well as the ratio performance to deviation (RPD) [30]. 

## 3. Results and Discussion

### 3.1. HPLC Reference Analysis of Piperine in Ground Black Pepper 

The ground black pepper (*n* = 160) used in the experiment was randomly divided into two sets: a calibration set (*n* = 120, 75%) and a prediction set (*n* = 40, 25%). The HPLC analysis showed that the piperine content ranged between 3.128 and 6.494 g/100 g (calibration set: 3.289 to 6.169 g/100 g, prediction set: 3.128 to 6.494 g/100 g), and the mean and the standard deviation were 4.689 and 0.599 g/100 g (calibration set) and 4.693 and 0.750 g/100 g (prediction set), respectively. Table 1 is a summary of statistical parameters from the analysis of piperine in ground black pepper. The chemical structure of piperine contains functional groups that are absorbed by the NIR spectroscopy. In addition, the piperine contents, mean and standard deviation were similar between the calibration and prediction sets, as shown in the result of HPLC reference analysis. HPLC analysis results were used as reference data in the chemometric model development using NIRS.

**Figure 1:**
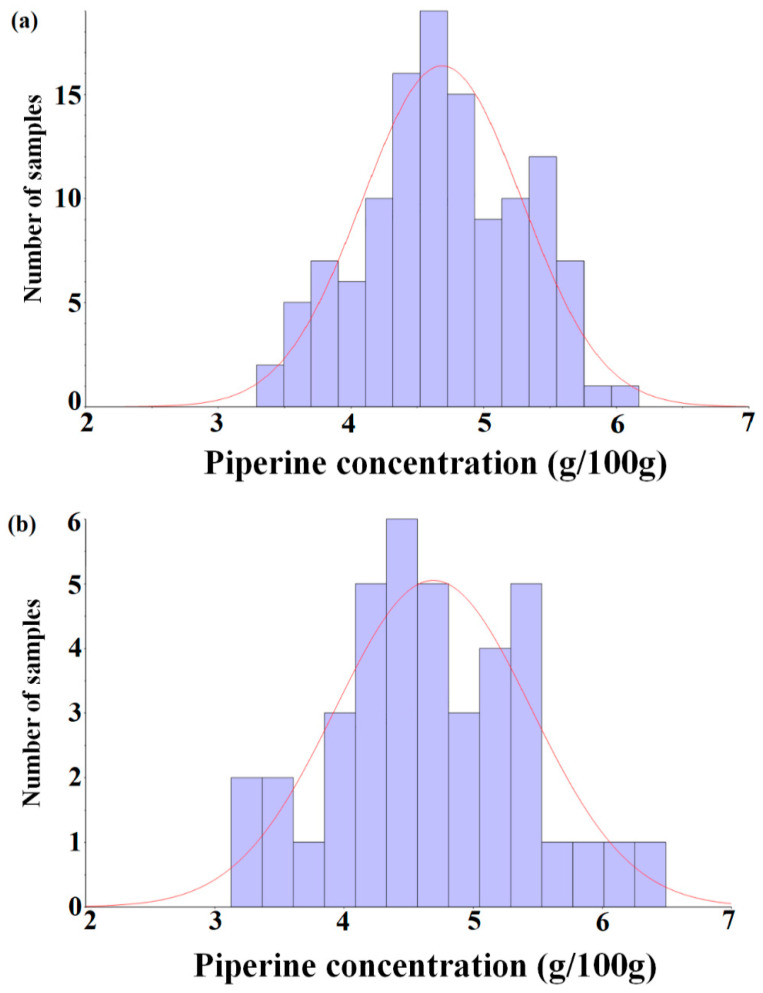
Histogram of the piperine concentration of the calibration set (*n* = 120, (**a**)) and prediction set (*n* = 40, (**b**)).

Figure 1 shows a histogram with respect to the distribution of piperine contents in the calibration and prediction sets. Since the purpose of this study is to develop an analysis method for the piperine content of ground black pepper using NIRS, the HPLC method was not further considered.

### 3.2. NIR Spectral Characteristics of P. nigrum and Spectra Pre-Treatment

**Figure 2:**
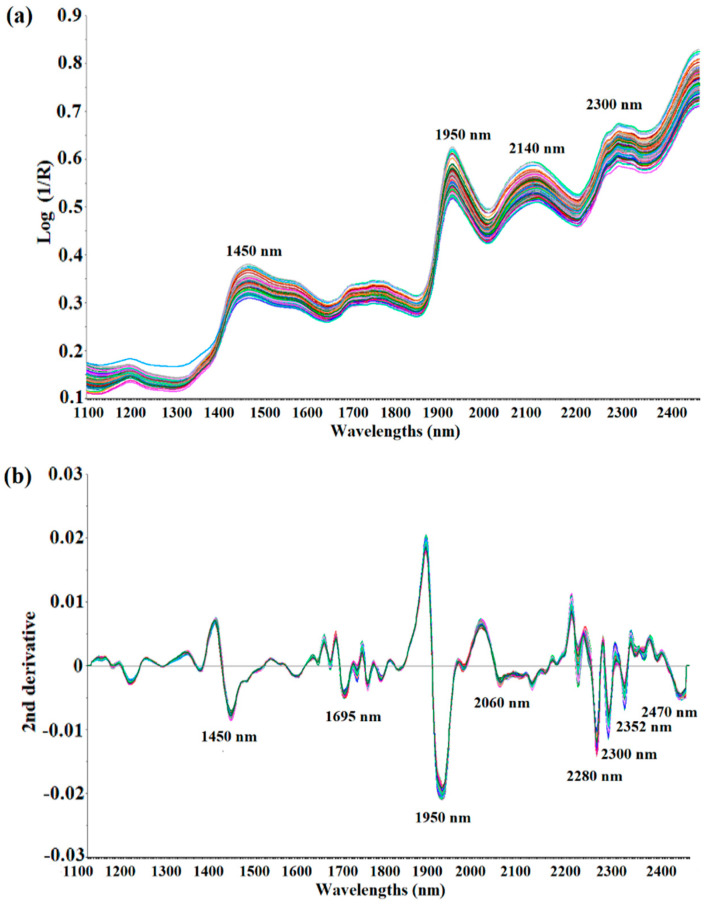
Raw spectra (**a**) and second derivative spectra treated with SNV-DT (**b**) of ground black pepper samples in NIR measurement.

Figure 2a shows the raw NIR spectra in the 1100 to 2500 nm wavelength range of the ground black pepper (*n* = 120) used for calibration modeling. The shapes of all 120 spectra were very similar, and about four wavelengths with strong absorption were observed. It was observed that strong absorption occurs near 1450 nm (O-H stretch, first overtone) and 1950 nm (C=O stretch, second overtone). Strong absorption was also determined between 2100 nm and 2300 nm, but it was difficult to establish the exact absorption wavelength as a combination band region over 2100 nm, therefore it was not possible to identify a specific functional group. The spectrum was pre-treated using de-trending (DT), SNV and derivation to obtain a sharper peak shape by minimizing interference from surrounding wavelengths. Figure 2b shows the spectra processed with the DT treatment + SNV + Savitzky–Golay second derivation (polynomial order 2, smoothing point 11) of the raw spectra. As a result of correcting the scattering and dispersion of light in each spectrum, and adjusting the baseline and derivation, the peak shape became sharper than that of the raw spectra, and significant absorption occurred at eight wavelengths, including 1695 nm (C-H stretch, first overtone), 2060 nm (N-H bend, second overtone or N-H bend/N-H stretch, combination), 2280 nm (C-H stretch/CH2, deformation), 2300 nm (C-H bend, second overtone), 2352 nm (CH2 bend, second overtone) and 2470 nm (C-H bend, combination) [31]. These raw NIR spectra of the ground black pepper samples contain O-H bonds, C-H bonds and N-H bonds, but it was difficult to perform quantitative analysis with respect to the molecular structure of piperine due to the overtone and combination of the NIRS spectra.

Therefore, a chemometrics model was designed to predict the exact piperine content of ground black pepper using the information of the NIR spectra after mathematical elaboration. 

### 3.3. Development of Chemometrics Models

De-trending (DT) and standard normal variate (SNV) transformation of the mathematical treatments were applied for a better correlation and prediction. The mathematical elaborations were performed with first and second order derivatives, and smoothing points 11 (left 5, center, right 5) and 21 (left 10, center, right 10), applying a Savitzky–Golay smoothing filter. 

The results of the partial least squares (PLS) regression modeling were the following: the *R*^2^ of the calibration set was in the range of 0.867 to 0.900, the root mean square error of calibration (RMSEC) was in the range from 0.190 to 0.218 g/100 g, and the standard error of calibration (SEC) was between 0.191 and 0.219 g/100 g. The *R*^2^ of the cross validation set was in the range of 0.848 to 0.869, the root mean square error of cross-validation (RMSECV) was between 0.218 and 0.236 g/100 g, and the standard error of cross-validation (SECV) ranged from 0.219 to 0.237 g/100 g. In modeling using PLS regression, the optimal conditions were a factor of 5, second derivative order and smoothing point 11. The results of the principal components (PC) regression chemometric modeling, *R*^2^, of the calibration set were confirmed to be in the range of 0.745–0.885, RMSEC was between 0.203 and 0.302 g/100 g, and SEC was in the range from 0.204 to 0.304 g/100 g. The *R*^2^ of the cross validation set was confirmed to be in the range of 0.733 to 0.868, RMSECV was between 0.219 and 0.312 g/100 g, and SECV ranged from 0.220 to 0.313 g/100 g. In the modeling using PC regression, the optimal conditions (conditions indicating high *R*^2^ and low RMSEC, SEC, RMSECV and SECV) were identified as factor 7, second derivative order and smoothing point 21 (Table 2).

**Figure 3:**
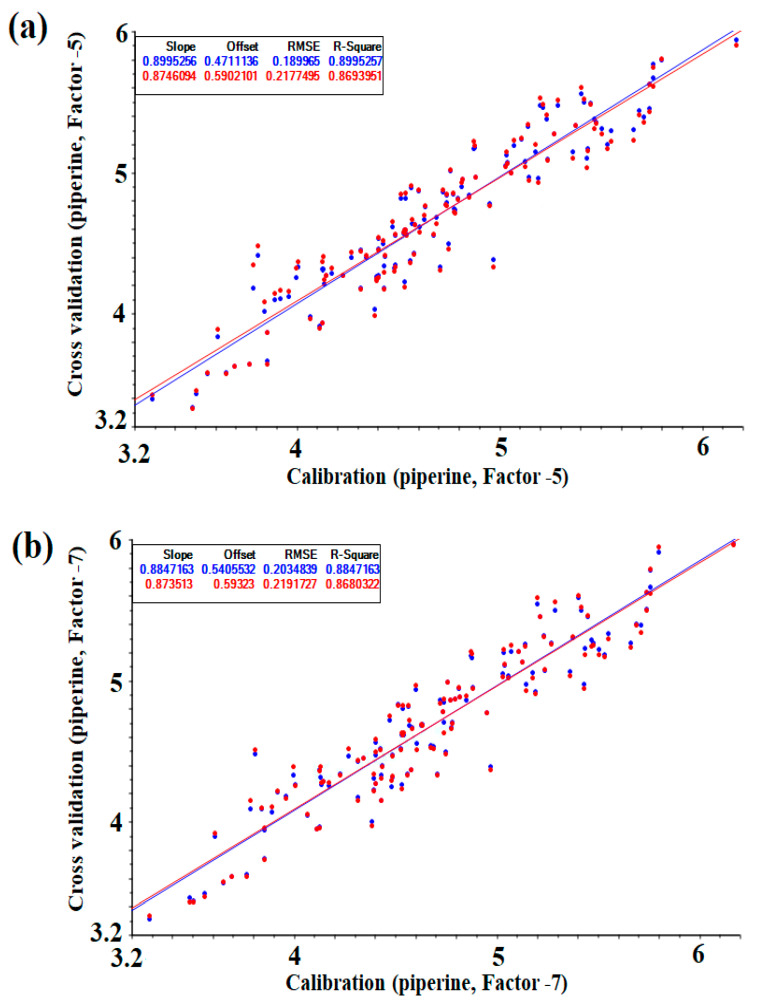
Correlations plot of calibration and cross-validation values versus NIR predicted by the optimized PLS and PC model (g/100): (**a**) PLS regression, (**b**) PC regression.

Figure 3 shows a correlation plot of the calibration and cross-validation sets for NIRS measurements of the reference values ((a) PLS, (b) PC). Comparing the PLS and PC values in the same mathematical treatment, the group modeled with PLS showed a better set of values (Table 2) and could be described as a calibration model without underfitting and overfitting with fever latent variables. However, there was no significant difference in the results at optimal conditions, and the prediction model was verified using both models.

### 3.4. Evaluation Parameters for Comparison between Cross-Validation and Prediction Set

**Figure 4:**
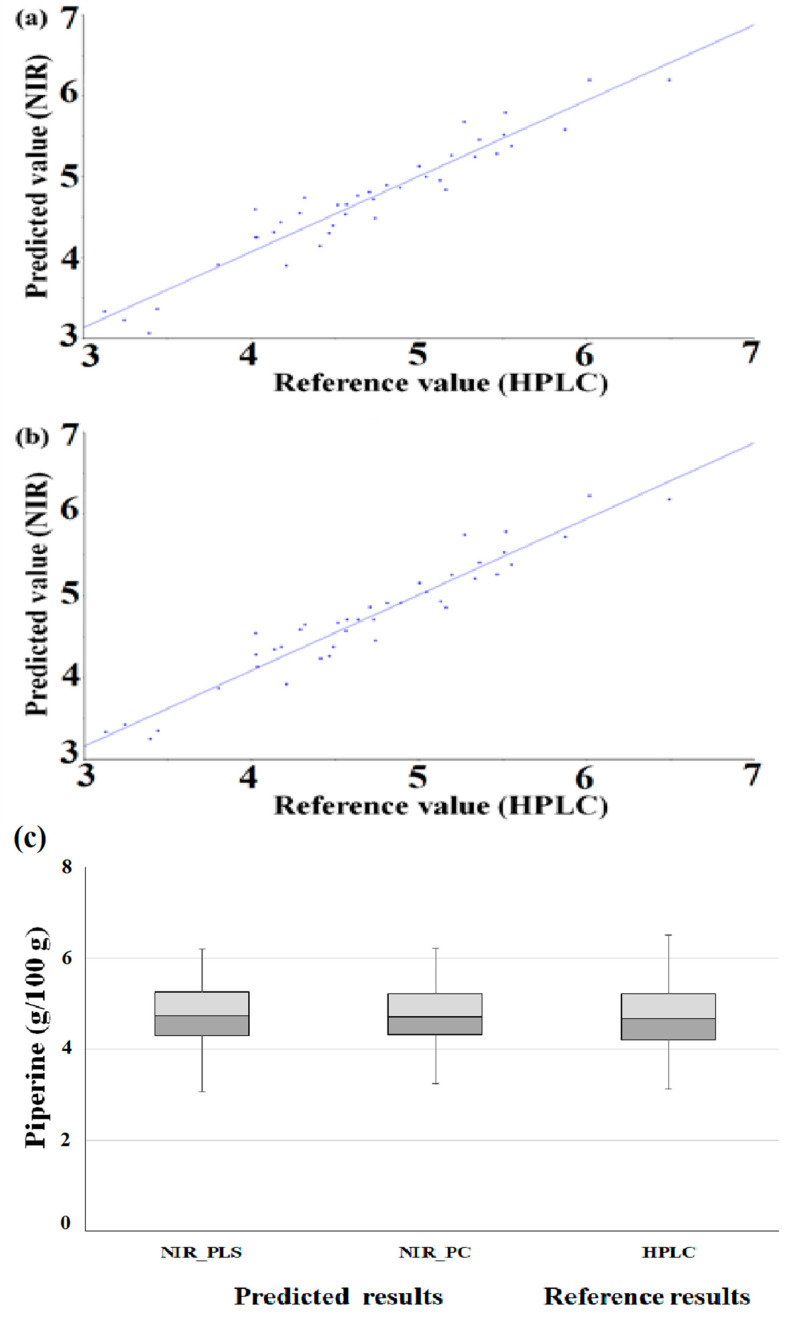
Method accuracy of developed regression (scatter plot of prediction model by optimized PLS (**a**) and PC (**b**) regression (g/100 g), box plot (**c**)).

After applying the prediction set to the optimized PLS and PC models, we evaluated the prediction against the cross-validation set. Figure 4 shows a correlation plot of reference values and prediction values ((a) PLS, (b) PC). 

In the PLS model, the prediction set had higher *R*^2^, RMSEP and SEP values compared to the *R*^2^, RMSECV and SECV of the cross-validation set. The prediction set had, compared to the cross-validation set, a good *R*^2^ value, and the RMSE and standard error values were also slightly higher. The RPD value was higher than that of the cross-validation set. It was confirmed that the standard deviation of the piperine content of the prediction set was larger than that of the calibration set, thereby improving the RPD value, which is the standard deviation divided by the standard error value. In the PC model, the prediction set had higher *R*^2^, RMSEP and SEP vales compared to the *R*^2^, RMSECV and SECV of the cross-validation set. The prediction set had excellent *R*^2^, RMSE and standard error values, and the RPD value was also higher than that of the cross-validation set (Table 3). The method accuracy of developed regression was evaluated with the box plot. As a result of the box plot of HPLC value and prediction value, the lower and upper quartiles and whiskers of similar ranges were identified (Figure 4c). 

Compared to the previous result of the piperine quantification in peppercorn studied by Schulz et al., the optimization of the calibration model was developed through various data-pretreatments in this study, and the statistical values of *R*^2^ and SEP were found to be superior to the existing research [32]. Furthermore, we compared these with NIR quantitative analysis results for other bioactive compounds using agricultural products. The results of quantifying the vitamin C content in apples were 0.80, 4.9 mg/100 g and 2.0 for *R*^2^, SEP and RPD, respectively [33], and those of quantifying the lycopene content in watermelons were 0.805, 16.19 mg/kg and 2.1 for *R*^2^, RMSEP and RPD, respectively [34]. When the total curcumin in turmeric was analyzed, *R*^2^ was 0.901, SEP was 0.067 g/100 g and RPD was 3.24 [35]. The *R*^2^ was > 0.8 and the RPD > 2.0, and the results of both the PLS and PC models obtained in this study were similar to those of other studies. In general, a higher RPD value indicates a better calibration model for an accurate prediction [36,37]. RPD values 1.5–2.0 show that the model can discriminate low from high values for the response variable. Rough quantitative predictions are possible when a value 2.0–2.5 is indicated, and a value in the range from 2.5 to 3 or above corresponds to an acceptable, up to excellent, prediction accuracy, respectively [38]. Moreover, Chang et al. (2001) defined an RPD < 1.4 as non-reliable, while a fair model is representative of 1.4 < RDP < 2.0, and an RPD > 2.0 is described as an excellent model [39]. Therefore, it was proven that the RPD value of 2.7 or higher (3.5 or higher for the prediction model) obtained in this study was sufficient reason to use NIRS instead of HPLC as a quantification tool for piperine. 

## 4. Conclusions

This study confirmed that the piperine in ground black pepper can be analyzed using NIRS. The NIRS analysis was conducted within 2 min per sample, compared to the HPLC analysis time (20 min) (this is just simply a comparison of the run time of instrument). Considering the time consumption of the pretreatment of the sample and the result interpretation of the HPLC method, NIRS was confirmed to be the more efficient analysis method.

After the spectra treatment, this research identified that the shape of the peak became sharper, and more absorption wavelengths occurred. The development of the PLS and PC regression chemometrics models using NIR spectra was achieved. The mathematical elaborations greatly affected the RMSE, RMSECV and SEP values of the developed chemometric models. In particular, the higher the derivative order, the lower the RMSE, RMSECV and SEP values were, as identified in both the PLS and PC models.

To obtain a better result, it was confirmed that optimization is required. As a result of optimization, the performance parameters of the PLS and PC regressions, according to derivative order, determined that the second derivative order is better than the first derivative order.

The developed model was verified using a prediction set, and high *R*^2^, low RMSEP and SEP and excellent RPD values were observed. Comparing the PLS and PC methods in the optimal model, it was shown that PC regression gives better results in the prediction set. However, it was suggested that the PLS model can describe a model developed with fewer latent variables. It was further verified that NIR is an economical and eco-friendly analysis method that has many advantages compared to HPLC, such as a simple pre-treatment process, short analysis time and no use of organic solvents, while obtaining similar results. These results show that NIRS can be applicable as a promising quality control tool to quantify piperine in *P. nigrum* L. (black pepper).

## Figures and Tables

**Table 1:** Statistics for piperine in ground black pepper for the calibration and prediction set of the reference method.

Compound	Parameters	Total Set (g/100 g)	Calibration Set (g/100 g)	Prediction Set (g/100 g)
Piperine	Number of Samples	160	120	40
	Min	3.128	3.289	3.128
	Max	6.494	6.169	6.494
	Mean	4.690	4.689	4.693
	S.D.	0.640	0.599	0.750

**Table 2:** Optimum calibration parameters according to partial least squares (PLS) and principal components (PC) regression analyses.

Regression	Factors	Math Elaboration		Calibration Set			Cross Validation Set		
		Derivative Order	Smoothing Point	*R* ^2 a^	RMSEC ^b^ (g/100 g)	SEC ^c^ (g/100 g)	*R* ^2^	RMSECV ^d^ (g/100 g)	SECV ^e^ (g/100 g)
PLS	4	1st	11	0.871	0.215	0.216	0.857	0.228	0.229
	4	1st	21	0.867	0.218	0.219	0.848	0.236	0.237
	**5**	**2nd**	**11**	**0.900**	**0.190**	**0.191**	**0.869**	**0.218**	**0.219**
	4	2nd	21	0.878	0.209	0.210	0.859	0.227	0.228
PC	3	1st	11	0.745	0.302	0.304	0.733	0.312	0.313
	7	1st	21	0.872	0.214	0.215	0.852	0.232	0.233
	7	2nd	11	0.878	0.210	0.210	0.863	0.224	0.225
	**7**	**2nd**	**21**	**0.885**	**0.203**	**0.204**	**0.868**	**0.219**	**0.220**

Figures in bold print represent optimum results; ^a^ *R*^2^: coefficient of multiple correlations in calibration; ^b^ RMSEC: root mean square of standard error of calibration; ^c^ SEC: standard error of calibration; ^d^ RMSECV: root mean square of standard error of cross-validation; ^e^ SECV: standard error of cross-validation (g/100 g).

**Table 3:** Statistics of cross-validation and prediction set for piperine by PLS and PC regression.

Regression	Cross-Validation Set				Prediction Set			
	*R* ^2^	RMSECV	SECV	RPD_CV_^a^	*R* ^2^	RMSEP ^b^	SEP ^c^	RPD_p_^d^
PLS	0.869	0.218	0.219	2.735	0.914	0.220	0.222	3.378
PC	0.868	0.219	0.220	2.723	0.921	0.210	0.210	3.571

^a^ RPD_CV_: the ratio of cross-validation to deviation (=SD_cal_/SECV); ^b^ RMSEP: root mean square of standard error of prediction; ^c^ SEP: standard error of prediction (g/100 g); ^d^ RPD_p_: the ratio of prediction to deviation (=SD_pre_/SEP).
